# Rechargeable Aqueous Zinc-Ion Batteries in MgSO_4_/ZnSO_4_ Hybrid Electrolytes

**DOI:** 10.1007/s40820-020-0385-7

**Published:** 2020-02-21

**Authors:** Yingmeng Zhang, Henan Li, Shaozhuan Huang, Shuang Fan, Lingna Sun, Bingbing Tian, Fuming Chen, Ye Wang, Yumeng Shi, Hui Ying Yang

**Affiliations:** 1 College of Chemistry and Environmental Engineering, Shenzhen University, Shenzhen, 518060 Guangdong People’s Republic of China; 2 International Collaborative Laboratory of 2D Materials for Optoelectronics Science and Technology of Ministry of Education, Institute of Microscale Optoelectronics, Shenzhen University, Shenzhen, 518060 People’s Republic of China; 3 Engineering Technology Research Center for 2D Material Information Function Devices and Systems of Guangdong Province, Shenzhen, People’s Republic of China; 4 Institute of Microscale Optoelectronics, Shenzhen University, Shenzhen, 518060 People’s Republic of China; 5 Pillar of Engineering Product Development, Singapore University of Technology and Design, 8 Somapah Road, Singapore, 487372 Singapore; 6 School of Physics and Telecommunication Engineering, South China Normal University, Guangzhou, 510006 People’s Republic of China; 7 Key Laboratory of Material Physics of Ministry of Education, School of Physics and Engineering, Zhengzhou University, Zhengzhou, 450052 People’s Republic of China

**Keywords:** Aqueous zinc-ion batteries, Hybrid electrolytes, Electrolyte additives, Magnesium ions, Vanadates

## Abstract

MgSO_4_ is chosen as an additive to address the capacity fading issue in the rechargeable zinc-ion battery system of Mg_x_V_2_O_5_·nH_2_O//ZnSO_4_//zinc. Electrolytes with different concentration ratios of ZnSO_4_ and MgSO_4_ are investigated. The batteries measured in the 1 M ZnSO_4_^−1^ M MgSO_4_ electrolyte outplay other competitors, which deliver a high specific capacity of 374 mAh g^−1^ at a current density of 100 mA g^−1^ and exhibit a competitive rate performance with the reversible capacity of 175 mAh g^−1^ at 5 A g^−1^. This study provides a promising route to improve the performance of vanadium-based cathodes for aqueous zinc-ion batteries with electrolyte optimization in cost-effective electrolytes.

**Electronic supplementary material:**

The online version of this article (10.1007/s40820-020-0385-7) contains supplementary material, which is available to authorized users.

## Highlights

- Divalent magnesium ions as electrolyte additives are first used to improve the performance of vanadium-based cathodes for aqueous ZIBs.
- Pre-adding magnesium ions into electrolytes provide an appropriate equilibrium balance between the dissolution and recombination of magnesium vanadates, thus suppress the continuous dissolution of active materials, and lead to a higher stability of the electrode.
- The hybrid aqueous electrolytes with cost-effective ZnSO_4_ and MgSO_4_ salts show a better competitive prospective for the stationary grid-scale applications.

**Electronic supplementary material:**

The online version of this article (10.1007/s40820-020-0385-7) contains supplementary material, which is available to authorized users.

## Graphical abstract

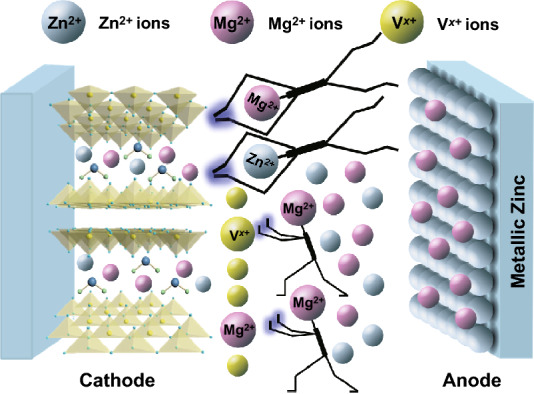

## Introduction

The growing demand for the commercial utilizations in grid-scale energy storage devices has inspired the continuing development of rechargeable systems, more considering the factors of low-cost, eco-friendly, and high operational safety [1, 2]. In these regards, rechargeable aqueous batteries based on earth-abundant Na, K, Al, Mg, Ca, and Zn are new promising alternatives [3–6] to the non-aqueous ones for stationary grid-scale applications, owing to their safer water-based electrolytes, higher ionic conductivity, and relatively lower cost [7, 8]. Among those different aqueous batteries, zinc-ion batteries (ZIBs) based on Zn^2+^ involved chemistry with a two-electron transfer mechanism are emerging as attractive technologies, which can directly employ high-capacity Zn metal as the anode materials and using mildly acidic or neutral aqueous electrolytes [8–10].

Up to now, the most intensively investigated cathode materials with outstanding performance for ZIBs are vanadium-based oxides with (de)intercalation-type storage mechanism and manganese-based oxides with reversible phase transition storage mechanism. Owing to their low cost and large interlayer spacing, metal vanadates and their derivatives have received tremendous attention. For instance, Nazar et al. constructed a vanadium oxide bronze (Zn_0.25_V_2_O_5_·nH_2_O) with a good rate performance [11]. Later, Mai’s group reported a Na_2_V_6_O_16_·1.63H_2_O nanowire cathode with a long cycling life [12]. Alshareef’s group also demonstrated layered Ca^2+^/Mg^2+^-intercalated V_2_O_5_ cathode materials (Ca_0.24_V_2_O_5_·nH_2_O [1] and Mg_0.34_V_2_O_5_·nH_2_O [13]) with a high capacity. Liang’s group further developed Ag_0.4_V_2_O_5_ [14] and several potassium vanadates [15] with different compositions for ZIBs.

Despite the booming development of ZIBs, there are still needs to pay more attention to the sufficient understanding and fundamental investigations of the Zn-ion storage mechanism. As we know, the electrolytes play major roles in forming protective layers on the cathode and anode surfaces and influence the formation of byproducts such as ZnO or Zn(OH)_2_ and basic zinc sulfate for ZIBs [16–18]. Therefore, the judicious choice and preparation of an appropriate electrolyte are as important as searching a promising cathode material for the long-term operation of ZIBs.

The vanadium-based as well as manganese-based electrode materials generally suffer from capacity fading upon cycling majorly due to the gradual dissolution of active materials into the electrolytes [16, 19]. For example, the Mn^2+^ dissolution from manganese-based oxides is caused by the disproportionation reaction of Mn^3+^ into Mn^4+^ and Mn^2+^ in the aqueous electrolytes [16, 20, 21]. Researchers realized that pre-adding proper metal ions into the electrolyte will provide an appropriate equilibrium balance between the dissolution and recombination of the active materials and thus suppress the continuous dissolution of such metal ions and lead to a high stability of the electrode [22–24]. For example, Oh et al. presented a pioneering report on the addition of MnSO_4_ in aqueous ZnSO_4_ electrolyte, which improved the reversibility of the cathodic reaction and suppressed the formation of basic zinc sulfate on the MnO_2_ electrode surface with the help of MnSO_4_ additive [22]. Recently, Liu et al. [23] and Chen et al. [24] further proved that long cycling stability of MnO_2_ will be obtained with the utilization of manganese(II) salts (MnSO_4_ or Mn(CF_3_SO_3_)_2_) as additives to the electrolytes. Similarly, metal vanadates also suffer from the inevitable dissolution of vanadium in the aqueous electrolytes, thus resulting in a decay of capacity during cycling performance. The insertion of metal ions (Na^+^, K^+^, Zn^2+^, Ca^2+^, Mg^2+^, etc.) [11–15] between the V_x_O_y_ layers can act as pillars to maintain the structural stability of the vanadates. If the inserted metal ions dissolve from the layered structure of the vanadates, free soluble vanadium species (e.g., VO^2+^) will be obtained [25], with the color of the electrolyte turning into yellow. Recently, Chen et al. found that the addition of Na_2_SO_4_ into the ZnSO_4_ electrolyte can balance the equilibrium and impede the continuous dissolution of Na^+^ from NaV_3_O_8_·1.5H_2_O [17]. Although the pioneering results show that the utilization of corresponding salts could prevent the continuous dissolution of active materials, the mechanism has not been totally understood and still needs to be further studied both experimentally and theoretically.

In this research, a facile and mild synthesis of Mg_x_V_2_O_5_·nH_2_O (MgVO) nanobelts is demonstrated and its application as a cathode material in aqueous ZIBs is further reported. The MgVO cathodes were investigated in five electrolytes with different concentration ratios of ZnSO_4_ and MgSO_4_, i.e., 2.0 M ZnSO_4_, 1.5 M ZnSO_4_–0.5M MgSO_4_, 1.0 M ZnSO_4_–1.0 M MgSO_4_, 0.5M ZnSO_4_–1.5 M MgSO_4_, and 2.0 M MgSO_4_ electrolytes. The electrochemical behaviors gradually change when increasing the MgSO_4_ concentration in the aqueous electrolytes. Comparing the electrochemical performance in five electrolytes, MgVO cathodes measured in the 1.0 M ZnSO_4_–1.0 M MgSO_4_ electrolyte obtain the best results, which deliver a high specific capacity of 374 mAh g^−1^ at a current density of 100 mA g^−1^, maintain a retention of 90.3% at a current density of 1 A g^−1^ for 200 cycles, and exhibit a reversible capacity of 175 mAh g^−1^ at 5 A g^−1^. As a result, optimized and cost-competitive aqueous electrolytes are found to achieve the improved electrochemical performance for aqueous ZIBs.

## Experimental Section

### Preparation of Mg_x_V_2_O_5_·nH_2_O Nanobelts (MgVO)

All the reagents used in this experiment were of analytical grade and directly used as received.

In a typical synthesis, 1 mmol of commercial V_2_O_5_ powder and 1 mmol of MgSO_4_ were dissolved in 10 mL of deionized water under vigorous stirring. After stirring for 24 h at 80 °C, the yellow solution turned into a dark-red powder precipitated from the solution. The precipitate was washed with water for several times to remove the impurities and then dried at 60 °C for 12 h.

### Materials Characterization

The as-synthesized samples were characterized by X-ray diffraction (XRD) on a Rigaku D/Max-2500 X-ray diffractometer (Japan) with a Cu Kα radiation source (*λ* = 0.15,418 nm) operated with an accelerating voltage of 40 kV. Field-emission scanning electron microscopy (FESEM) measurements were conducted on a scanning electron microscope (Hitachi SU8010, Japan) operated with an accelerating voltage of 5 kV. The EDS elemental mapping data are obtained on the high-resolution scanning electron microscope (FEI APREO S). High-resolution transmission electron microscope (HRTEM) images and the selected area electron diffraction (SAED) patterns were taken on a JEOL JEM-2100 (Japan) transmission electron microscope performed with an accelerating voltage of 200 kV. The X-ray photoelectron spectroscopy (XPS) measurements were conducted on a K-Alpha + spectrometer (ThermoFisher Scientific, East Grinstead, UK). In situ XRD measurements were performed on a Bruker D8 Advance X-ray diffractometer (Germany) with a stainless-steel Swagelok-type cell that was connected to the Neware battery testing system (China).

### Electrochemical Measurements

#### Preparation of Electrolytes with Different Concentrations of ZnSO_4_ and MgSO_4_

In the series of measurements, five comparable electrolytes were used with different concentrations of ZnSO_4_ and MgSO_4_ in the aqueous solutions. The detailed concentration variations of ZnSO_4_ and MgSO_4_ are listed below:

1. 2.0 M ZnSO_4_ (2Zn0Mg) electrolyte: 2 mol L^−1^ of ZnSO_4_ aqueous solution;
2. hybrid 1.5 M ZnSO_4_–0.5M MgSO_4_ (1.5Zn0.5Mg) electrolyte: aqueous solution with 1.5 mol L^−1^ of ZnSO_4_ and 0.5 mol L^−1^ of MgSO_4_;
3. hybrid 1.0 M ZnSO_4_–1.0 M MgSO_4_ (1.0Zn1.0Mg) electrolyte: aqueous solution with 1.0 mol L^−1^ of ZnSO_4_ and 1.0 mol L^−1^ of MgSO_4_;
4. hybrid 0.5M ZnSO_4_–1.5 M MgSO_4_ (0.5Zn1.5Mg) electrolyte: aqueous solution with 0.5 mol L^−1^ of ZnSO_4_ and 1.5 mol L^−1^ of MgSO_4_;
5. 2.0 M MgSO_4_ (0Zn2Mg) electrolyte: 2 mol L^−1^ of MgSO_4_ aqueous solution.

#### Coin-Type Cell Assembling

The working electrode was fabricated by mixing vanadate nanobelts (70 wt%) as the active material, with Super P (20 wt%) as the conductive material, and polytetrafluoroethylene (10 wt%) as the binder. The electrode was fabricated by dropping the mixed slurry into the carbon paper and the active material was then absorbed onto the carbon fibers. The carbon paper was bought from Toray Industries, Inc. (Japan, TGP-H-090), and the thickness of the carbon paper was 0.28 mm. The electrode was cut into a circular disk (*d* = 11 mm) with the area of about 1.0 cm^2^, and the mass loading is about 0.7 mg cm^−2^. To evaluate the electrochemical performance, the battery testing was conducted in 2032 coin-type cells with the above aqueous solution as the electrolyte, glass fiber filter as a separator, and Zn metal foil as an anode.

#### Electrochemical Testing

Galvanostatic charge–discharge measurements were conducted on a multi-channel battery analyzer (Neware battery testing system, Shenzhen Neware Electronic Co., Ltd) at different current densities in the potential window between 0.2 and 1.4 V at room temperature (~ 25 °C). The cyclic voltammetry (CV) tests were performed on an electrochemical workstation (Autolab/M204, Metrohm, Switzerland) in the voltage range (1.4–0.2 V vs Zn/Zn^2+^) at room temperature at a scan rate of 0.1, 0.2, 0.3, 0.4, and 0.5 mV s^−1^. The electrochemical impedance spectroscopy (EIS) also performed on the above workstation in a frequency range 0.01–100 kHz at open-circuit voltage.

### In Situ XRD Measurements

In situ XRD measurements were performed in the 1.0 M ZnSO_4_–1.0 M MgSO_4_ electrolyte at current density of 100 mA g^−1^ within a potential window from 0.2 to 1.4 V. The electrode was assembled with a stainless-steel Swagelok-type cell that was connected to the Neware battery testing system (China). In contrast to conventional coin-cell setup, the specialized setup was utilized to ensure high sensitivity through the use of a polymide window and thin carbon paper support as a current collector. Herein, the polymide was used for the X-ray window materials instead of the generally used Beryllium (Be), because the Be metal will suffer from corrosion in the acidic aqueous solution.

## Results and Discussion

### Characterization of the MgVO Nanobelts

**Fig. 1:**
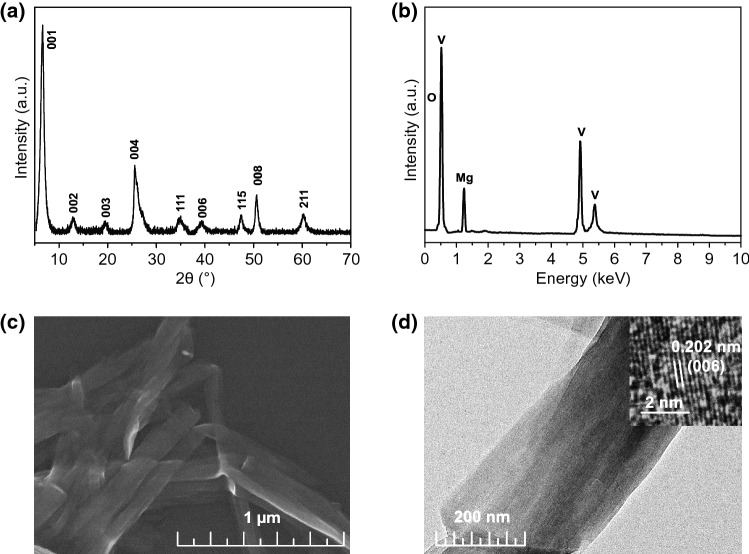
Structural characterization of MgVO cathodes: **a** XRD pattern, **b** EDS spectrum, **c** SEM image, **d** TEM image

The MgVO nanobelts were prepared by a facile one-pot synthesis. The structural characterization of the as-prepared MgVO was investigated by XRD, XPS, FESEM, and TEM. As shown in Fig. 1a, the typical XRD pattern is dominated by (00l) reflections, indicating that the preferred orientation along the *c* axis is to a high degree. Essentially, the MgVO compound is originated from the parent material of V_2_O_5_·1.6 H_2_O (JCPDS NO. 40-1296), which is completely different from the crystal structure of the commercial V_2_O_5_ reagent (Fig. S1). The diffraction peaks are strong and narrow, which indicates the crystallinity is as good as the ones obtained by hydrothermal methods [13]. The (001) peak located at 2*θ* = 6.34° corresponded to a larger interlayer spacing of 13.93 Å. The enlarged spacing would allow fast and efficient Zn^2+^ insertion, thus leading to a high rate performance for ZIBs.

The elements in MgVO are identified by EDS in Fig. 1b and further confirmed the existence of Mg, V, and O in MgVO by XPS in Fig. S2, in which the Mg, V, and O elements with the mole ratio of Mg/V closing to 1:8 are identified. The typical morphology of MgVO shown in FESEM image indicates the good uniformity of the product (Fig. 1c), which is composed of nanobelts with a general size of several micrometers in length and ~ 200 nm in width. The nanobelt-shaped characteristic is evidenced by the existence of several twisted belts in Fig. 1c. As shown in Fig. 1d, the TEM image further confirms the nanobelt structure, which is consistent with the FESEM observation. High-resolution transmission electron microscope (HRTEM) displays the marked lattice fringe with spacing of 0.202 nm, which is corresponded to the (006) plane of MgVO.

### Electrochemical Behaviors of the MgVO Electrodes in Five Different Electrolytes

**Fig. 2:**
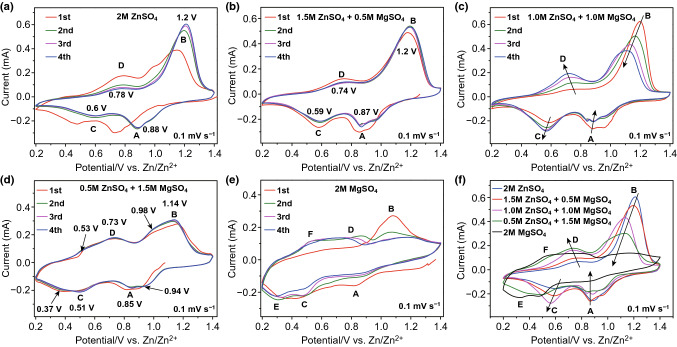
CV profiles for MgVO cathode at scan rate of 0.1 mV s^−1^ in different electrolytes: **a** 2.0 M ZnSO_4_, **b** 1.5 M ZnSO_4_–0.5 M MgSO_4_, **c** 1.0 M ZnSO_4_–1.0 M MgSO_4_, **d** 0.5 M ZnSO_4_–1.5 M MgSO_4_, **e** 2.0 M MgSO_4_, and **f** the comparison of the third CV profiles in the five electrolytes

The electrochemical behaviors of ions insertion/extraction in MgVO were investigated in five types of zinc/magnesium salt electrolytes with different concentration ratios. The initial four cycles of cyclic voltammetry (CV) plots for the MgVO electrode examined in the five types of electrolytes within a potential window from 0.2 to 1.4 V at a scan speed of 0.1 mV s^−1^ are, respectively, shown in Fig. 2.

In the single 2.0 M ZnSO_4_ electrolyte, two redox couples are observed for the MgVO cathode (Fig. 2a). One redox couple at ~ 0.6/0.78 V shows a pair of broad reduction/oxidation peaks with comparable intensity, while the other redox couple at ~ 0.88/1.2 V shows a broad cathodic peak with a small shoulder (0.96 V) and an intensive distinct anodic peak. In the negative scan, the cathodic peaks demonstrate the electrochemical intercalation of Zn^2+^ ions into the layered structure of MgVO, corresponding to the continuous valence changes of vanadium from V^5+^ to lower oxidation states [17]. In the reverse positive scan, the anodic peaks are attributed to the corresponding extraction of Zn^2+^ ions from the layered MgVO framework, indicating the converse valence changes of vanadium may occur in the reverse scan. The shifting of the reduction/oxidation peaks in the first cycle could be related to the gradual activation of the fresh electrode and might correspond to the process that most of the Mg^2+^ ions in the MgVO-layered structure has been deintercalated during the first positive scan and further replaced by Zn^2+^ ions to form a Zn^2+^/Mg^2+^ combining vanadates which serves as the following cathode material in the subsequent cycles [13]. Such a phenomenon can be explained by the displacement–intercalation reaction mechanism, which has been observed in Ag_0.4_V_2_O_5_ [14] and Mg_0.34_V_2_O_5_·0.84H_2_O [13] for rechargeable aqueous ZIBs and this storage mechanism has also been observed in lithium-ion batteries [26, 27].

In Fig. 2b, one-fourth of the zinc salt was replaced by MgSO_4_ to obtain a mixed 1.5 M ZnSO_4_–0.5M MgSO_4_ (1.5Zn0.5Mg) electrolyte. The insertion/extraction behaviors of the electrochemically activated ions in MgVO are very similar to the pure 2.0 M ZnSO_4_ case (Fig. 2a). There are two pairs of redox peaks located at 0.59/0.74 and 0.87/1.2 V. In the negative scan, the cathodic peaks at 0.59 and 0.87 V with an obvious shoulder (0.96 V) demonstrate the electrochemical reductions of vanadium from V^5+^ to lower oxidation states [17], corresponding to the insertion process of activated cations into the MgVO-layered structure. In the reverse positive scan, a broad anodic peak at around 0.74 V and an intensive anodic peak at 1.2 V indicate the converse oxidations of vanadium valences, corresponding to the extraction of activated ions from the lattice of the MgVO cathode. It is noted that after the first cycle, subsequent three cycles show a nearly overlapping shape, indicating the better reversibility of cation intercalation/deintercalation. The peak difference of the CV curves between the 2Zn0Mg and 1.5Zn0.5Mg electrolytes will be shown clearly from the comparison in Fig. 2f.

In the hybrid 1.0Zn1.0Mg electrolyte with mixed 1.0 M ZnSO_4_ and 1.0 M MgSO_4_ solution (Fig. 2c), the electrochemical behavior of cation insertion/extraction in the MgVO cathode seems different from that in the 2Zn0Mg and 1.5Zn0.5Mg electrolytes. Notably, from the first to the fourth cycle, the redox couple of reduction peak C and oxidation peak D shifts to lower voltage with intensity increased gradually. More obviously, it is noted that the distinct anodic peak which originally appeared at 1.2 V shifts to a lower voltage (at about 1.1 V) and decreases the intensity significantly with the increase in cycles. From the fourth cycle, it is observed that the shape of the oxidation peak B also changes slightly to a wider one, and the intensity ratio of the two oxidation peaks (B/D) tends to become smaller than the different cases in the 2Zn0Mg and 1.5Zn0.5Mg electrolytes (Fig. 2a, b). The changes of the electrochemical behaviors are further discussed and confirmed by the CV profiles tested in the 0.5Zn1.5Mg and 2.0 M MgSO_4_ electrolytes (Fig. 2d, e).

As the concentration of MgSO_4_ increased to 1.5 mol L^−1^, a hybrid 0.5 M ZnSO_4_–1.5 M MgSO_4_ electrolyte was obtained. The corresponding CV scans presented in Fig. 2d show two pairs of reduction/oxidation peaks (0.51/0.73 V and 0.85/1.14 V); each of them displays a shoulder located at 0.37, 0.53, 0.94, and 0.98 V, respectively. The newly appeared shoulder peaks could be attributed to the insertion/extraction processes of magnesium ions into/from the layered structure of MgVO in the Mg^2+^ containing solution. The Mg^2+^ co-insertion phenomenon in the aqueous ZIBs was recently reported in the Mg_0.34_V_2_O_5_·0.84H_2_O//Zn(CF_3_SO_3_)_2_ electrolyte//Zinc system [13]. The deintercalated Mg^2+^ ions were found to deposit on the Zn anode upon charging process, then dissolve back in the electrolyte and serve as the co-insertion cations along with the Zn^2+^ ions upon discharge [13]. As a result, the reduction/oxidation peaks with shoulders reflect the insertion/extraction processes of both Zn^2+^/Mg^2+^ cations coexist.

In contrast, the CV curves in the case of 2.0 M MgSO_4_ electrolyte without ZnSO_4_ are significantly different from the ones in the ZnSO_4_ containing solutions (Fig. 2e). The original redox couple below 0.8 V split into two pairs of reduction/oxidation peaks (C/D and E/F peaks), while the original redox couple above 0.8 V becomes less and less obvious. The new cathodic peak E appeared at nearly 0.3 V and the corresponding anodic peak F appeared at nearly 0.5 V could be resulted from the shifting of the shoulders in the reduction/oxidation (C/D) peaks. This result further confirms that the donor and acceptor of electrons accompany with the (de)intercalation process of magnesium ions into the lattice of the MgVO-layered structure in the MgSO_4_ electrolyte.

Figure 2f presents the comparison of CV curves in the different five electrolytes at the third cycle, which shows clearly the difference of the peaks when the concentration of MgSO_4_ increases from zero to 2.0 mol L^−1^. It is obviously seen that the distinct intensive anodic peaks B shift to lower voltage gradually and the intensity decreases significantly with the increase in the MgSO_4_ concentration. The intensity of the redox couple of reduction peak C and oxidation peak D gradually increases in an order of 1.0Zn1.0Mg > 1.5Zn0.5Mg > 2Zn0Mg. The newly appeared peaks/shoulders and the peak shifting are also distinguished in the case of the 0.5Zn1.5Mg and 2.0 M MgSO_4_ electrolytes.

**Fig. 3:**
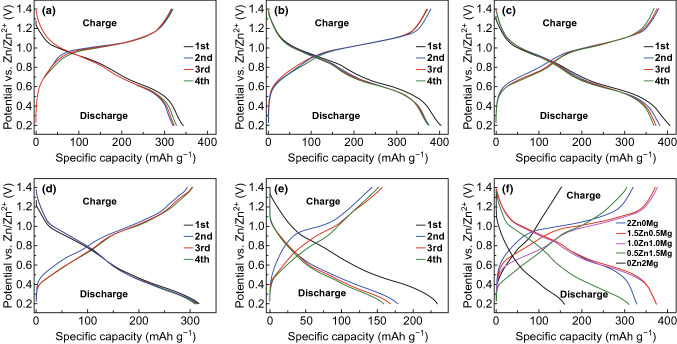
Discharge–charge profiles for MgVO cathode at current density of 100 mA g^−1^ in different electrolytes: **a** 2.0 M ZnSO_4_, **b** 1.5 M ZnSO_4_–0.5 M MgSO_4_, **c** 1.0 M ZnSO_4_–1.0 M MgSO_4_, **d** 0.5 M ZnSO_4_–1.5 M MgSO_4_, **e** 2.0 M MgSO_4_, and f the comparison of the fourth discharge–charge profiles in the five electrolytes

The insertion and deinsertion of Zn^2+^/Mg^2+^ cations into/from the MgVO-layered structure are further evaluated by galvanostatic discharge and charge tests in a potential range from 0.2 to 1.4 V at different current densities. The galvanostatic discharge–charge measurement results at current density of 100 mA g^−1^ with initial four curves in five different electrolytes, i.e., 2Zn0Mg, hybrid 1.5Zn0.5Mg, hybrid 1.0Zn1.0Mg, hybrid 0.5Zn1.5Mg, and 0Zn2Mg, are, respectively, illustrated in Fig. 3. In general, the distinct discharge and charge profiles observed in the 2.0 M ZnSO_4_ (Fig. 3a), 1.5Zn0.5Mg (Fig. 3b), and 1.0Zn1.0Mg (Fig. 3c) electrolytes show some similar electrochemical behavior, while curves in 0.5Zn1.5Mg (Fig. 3d) and 2.0 M MgSO_4_ (Fig. 3e) electrolytes exhibit an obvious difference.

In the pure 2.0 M ZnSO_4_ electrolyte (Fig. 3a), two potential plateaus appear at about 0.9 and 0.6 V during the discharge process. In the charge curves, an obvious plateau near 1.0 V can be observed with a steep slope in the potential range from 0.7 to 0.9 V. The initial discharge capacity is 343 mAh g^−1^ with a charge capacity of 316 mAh g^−1^, the corresponding initial coulombic efficiency is about 92.1%. In the hybrid 1.5Zn0.5Mg electrolyte with one-fourth of the zinc salt was replaced by MgSO_4_ (Fig. 3b), a larger first discharge capacity of 403 mAh g^−1^ is presented and the corresponding charge capacity is as high as 378 mAh g^−1^ with an initial coulombic efficiency of 93.8%. Similarly, for the hybrid 1.0Zn1.0Mg electrolyte, a high discharge capacity of 405 mAh g^−1^ is initially obtained of which a charge capacity of 379 mAh g^−1^ is recovered upon charging and the corresponding initial coulombic efficiency is about 93.6% (Fig. 3c). Comparing the above three curves shown in Fig. 3f, it can be observed that the discharge plateau near 0.6 V becomes more obvious and broader, corresponding to the gradual increase in reduction peak intensity in the CV results (Fig. 2f). Furthermore, during the charge process, the potential plateau appearing at about 1.0 V becomes narrower, while the potential slope in the range from 0.7 to 0.9 V tends to gradually become a plateau with the increase in the MgSO_4_ concentration. To make the plateau changes more obviously, the discharge and charge profiles are transformed to the corresponding differential capacity (dQ/dV) curves (Fig. S3), which are very similar to the CV results (Fig. 2). Increasing the concentration of MgSO_4_ to 1.5 mol L^−1^, the discharge–charge curves in the 0.5Zn1.5Mg electrolyte (Fig. 3d) starts to show the difference, of which the potential plateaus are less obvious and tend to become slops. In contrast, the discharge–charge profiles in the case of 2.0 M MgSO_4_ electrolyte exhibit a predominant slope without obvious plateaus (Fig. 3e).

The cycling performance measurements of Zn anodes in symmetric cells have firstly been done to investigate the influence of these hybrid electrolytes. As shown in Fig. S4, the polarization tests of symmetric Zn/electrolyte/Zn cells are reflected by the zinc plating/stripping experiments at a current density of 10 mA cm^−2^ for 5000 cycles. Comparing the overpotentials of the symmetric Zn/electrolyte/Zn cells in five different electrolytes, it can be seen that the overpotentials for the symmetric cells conducting in hybrid electrolytes (1.5 M ZnSO_4_–0.5 M MgSO_4_ (Fig. S4b), 1.0 M ZnSO_4_–1.0 M MgSO_4_ (Fig. S4c), 0.5 M ZnSO_4_–1.5 M MgSO_4_ (Fig. S4d)) are stabilized in the range of 0.2 V during the whole plating/stripping process. By contrast, the Zn/2.0 M ZnSO_4_/Zn cell (Fig. S4a) shows some unstable voltage polarization during the cycling measurement, while the Zn/2.0 M MgSO_4_/Zn cell (Fig. S4e) presents an overpotential value reached to 0.8 V after 5 h of cycling. The polarization testing results indicate that the appropriate pre-addition of magnesium ions can provide a more stable zinc plating/stripping cycling reversibility.

**Fig. 4:**
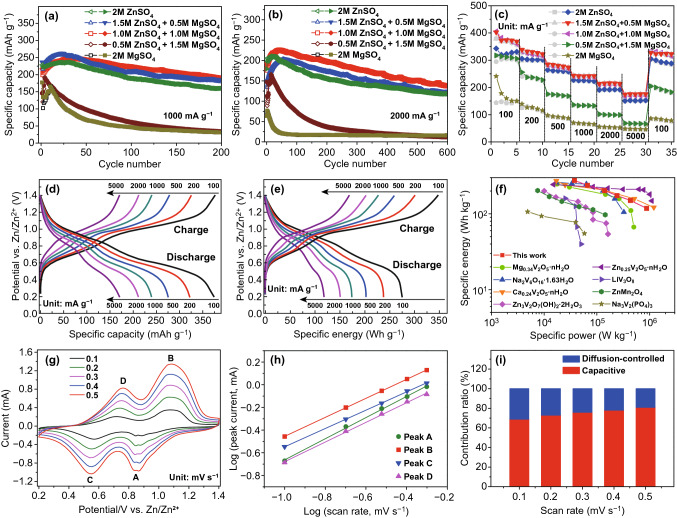
Zn-ion storage performance of the as-obtained MgVO cathodes. Cycling performance at current density of **a** 1000 and **b** 2000 mA g^−1^ in different electrolytes. **c** Rate performance at various rates in five electrolytes. **d** Rate discharge–charge curves in 1.0 M ZnSO_4_–1.0 M MgSO_4_ electrolyte and **e** the corresponding specific energies at each current density. **f** The Ragone plot of Zn//MgVO cell with 1.0 M ZnSO_4_–1.0 M MgSO_4_ electrolyte, in comparison with other aqueous ZIBs: Mg_0.34_V_2_O_5_·nH_2_O [13], Na_2_V_6_O_16_·1.63H_2_O [12], Ca_0.24_V_2_O_5_·nH_2_O [1], Zn_3_V_2_O_7_(OH)_2_·2H_2_O [31], Zn_0.25_V_2_O_5_·nH_2_O [11], LiV_3_O_8_ [32], ZnMn_2_O_4_ [10], Na_3_V_2_(PO_4_)_3_ [19].** g** CV curves at different scan rates in 1.0 M ZnSO_4_–1.0 M MgSO_4_ electrolyte and **h** the corresponding log(current) versus log(scan rate) plots of four peaks in the CV curves. **i** The capacitive contributions at different scan rates in 1.0 M ZnSO_4_–1.0 M MgSO_4_ electrolyte

Cycling performance of the MgVO electrodes is investigated in five electrolytes of 2Zn0Mg, hybrid 1.5Zn0.5Mg, hybrid 1.0Zn1.0Mg, hybrid 0.5Zn1.5Mg, and 0Zn2Mg, respectively, with the current densities of 1 and 2 A g^−1^ (Fig. 4a, b). It can be observed that the cycle plots can be divided into two groups, the group (I) with good cycling stability is measured in the 2Zn0Mg, 1.5Zn0.5Mg, and 1.0Zn1.0Mg electrolytes, while the other group (II) tested in the 0.5Zn1.5Mg and 0Zn2Mg electrolytes shows an obvious capacity fading. Besides, it can also be realized that the specific capacity of each system increases over several cycles until to a maximum value (the first 50 cycles for group I, and the first 10 cycles for group II), indicating that an activation is required to improve the kinetics and the discharge capacity then can be fully displayed [13, 28–30].

At a current density of 1 A g^−1^ (Fig. 4a), the electrode in the 1.0Zn1.0Mg electrolyte exhibits the slowest capacity fading and the highest capacity retention, of which a retention of 90.3% with a fading of about 10% can be achieved for 200 cycles. The electrode in the 1.5Zn0.5Mg electrolyte exhibits the highest capacity in the initial 40 cycles with a capacity retention of 81.8% over 200 cycles, while a lower capacity retention of 71.7% was obtained for the 2Zn0Mg electrolyte. When the current density further increases to 2 A g^−1^ (Fig. 4b), the electrode in the 1.5Zn0.5Mg electrolyte shows a lower starting specific capacity and leads to the highest capacity retention of 84% for the prolonged cycling of 600 cycles. The electrode in the 1.0Zn1.0Mg electrolyte exhibits the highest specific capacity over the whole 600 cycles, with a capacity retention of 72.8%. Similarly to that of the current density of 1 A g^−1^, a lower capacity retention of 58.9% was obtained for the electrode in the 2Zn0Mg electrolyte. Obviously, the cycling performance of group I shows a comparable advantage over the group II. Besides, prolonged cycling performance of 2500 cycles in the five different electrolytes with the current density of 5000 mA g^−1^ is shown in Fig. S5, which further presents the MgVO electrode in the 1.0Zn1.0Mg electrolyte exhibits the slowest capacity fading. For comparison, the electrochemical performance of the commercial V_2_O_5_ powders has also been evaluated and shown in Fig. S6.

Rate performance at various rates of the MgVO electrodes in five different electrolytes is also evaluated. As shown in Fig. 4c, comparing to the 2Zn0Mg electrode, the electrodes in the 1.5Zn0.5Mg and the 1.0Zn1.0Mg electrolytes exhibit relatively higher capacities, which deliver an average capacity of 374, 330, 280, 241, 216, and 175 mAh g^−1^ at a current density of 100 mA g^−1^, 200 mA g^−1^, 500 mA g^−1^, 1 A g^−1^, 2 A g^−1^, and 5 A g^−1^, respectively. When the current density further recovers to 100 mA g^−1^, the reversible capacity can recover back to 332 mAh g^−1^, suggesting that the MgVO electrode possesses excellent rate capability. While the 2Zn0Mg electrode delivers an average capacity of 322, 301, 261, 225, 191, and 151 mAh g^−1^ at a current density of 0.1, 0.2, 0.5, 1.0, 2.0, and 5.0 A g^−1^, respectively. When the rate recovers to 100 mA g^−1^, the reversible capacity of the 2Zn0Mg electrode can retain at 290 mAh g^−1^. Besides, an average capacity of 311, 236, 172, 133, 99, and 66 mAh g^−1^ for the 0.5Zn1.5Mg electrode and 160, 126, 92, 67, 53, and 47 mAh g^−1^ for the 0Zn2Mg electrode are also obtained at various rates.

The rate performance and the specific capacity at low current density of the MgVO electrodes are comparable to the results of metal vanadates recently reported in the literature. For example, Mg_0.34_V_2_O_5_·nH_2_O nanobelts were hydrothermally synthesized at 220 °C for 48 h, which exhibited the rate performance of 81 mAh g^−1^ at 5 A g^−1^ and about 353 mAh g^−1^ at a current density of 50 mA g^−1^ for ZIBs [13]. Another recent work reported a Na_2_V_6_O_16_·1.63H_2_O nanowire cathode prepared by hydrothermally treating at 180 °C for 24 h, which exhibited a specific capacity of 352 mAh g^−1^ at 50 mA g^−1^ and a rate performance of 219 mAh g^−1^ at 1 A g^−1^ and 162 mAh g^−1^ at 2 A g^−1^ [12]. An earlier report shows that the Zn_3_V_2_O_7_(OH)_2_·2H_2_O nanowires, obtained by microwave synthesis at 180 °C for 6 h, delivered a specific capacity of 213 mAh g^−1^ at a current density of 50 mA g^−1^ and a rate performance of 84 mAh g^−1^ at 1 A g^−1^ and 75 mAh g^−1^ at 2 A g^−1^ [31]. The detailed comparison of electrochemical properties of MgVO with previously reported metal vanadate cathodes is presented in Table S1.

Figure 4d displays the discharge–charge curves at different current rates in the 1.0 M ZnSO_4_–1.0 M MgSO_4_ electrolyte and the corresponding specific energies at each current density can also be obtained (Fig. 4e). This MgVO electrode delivers a specific energy of 275 Wh kg^−1^ at the specific power of 37,400 W kg^−1^ based on the mass of the cathode material. The energy loss for the MgVO material is low with high specific energies of 150 and 118 Wh kg^−1^ at specific power values as high as 432,000 and 875,000 W kg^−1^ are achieved, respectively. The Ragone plots shown in Fig. 4f further reflect that the performance of the MgVO cathode competes well with those of other reported cathodes for aqueous ZIBs, which exhibits a relatively low energy loss among the cathodes of Mg_0.34_V_2_O_5_·nH_2_O [13], Na_2_V_6_O_16_·1.63H_2_O [12], Ca_0.24_V_2_O_5_·nH_2_O [1], Zn_3_V_2_O_7_(OH)_2_·2H_2_O [31], Zn_0.25_V_2_O_5_·nH_2_O [11], LiV_3_O_8_ [32], ZnMn_2_O_4_ [10], Na_3_V_2_(PO_4_)_3_ [19] reported so far.

The EIS measurements are also conducted to investigate the charge transfer kinetics of the MgVO electrodes tested in these five different electrolytes (Fig. S7). The EIS measurements of the five electrodes were carried out in the frequency range of 0.01–100 kHz, after cycled at a current density of 100 mA g^−1^ for 10 cycles. The inset shows the corresponding equivalent EIS circuitry model, where *R*_s_ and *R*_ct_ represent the resistances of current collector/electrolyte, and the charge transfer, respectively. And the CPE and *Z*_w_ are the double-layer capacitance and Warburg impedance, respectively. As shown in Fig. S7a, the resulting Nyquist plots of the MgVO electrodes in all five electrolytes are composed of a semicircle in the high-frequency range with an extended linear tail in the low-frequency region, which are related to the charge transfer resistance (*R*_ct_) and the Warburg impedance of the ion diffusion in the solid materials, respectively. In the high-frequency regions, the MgVO electrode in hybrid 1.5Zn0.5Mg and 1.0Zn1.0Mg electrolyte features smaller semicircle in comparison to that of the electrodes in other electrolytes, corresponding to a lower charge transfer resistance. The *R*_ct_ value distinguished for the 1.5Zn0.5Mg and 1.0Zn1.0Mg system is 78 and 106 Ω, respectively. By contrast, the other electrolyte systems have bigger *R*_ct_ values, which are 275 (2Zn0Mg), 214 (0.5Zn1.5Mg), and 334 Ω (0Zn2Mg), respectively.

As we all known, the diagonal lines in the low-frequency region presented in the EIS results are assigned to the ion diffusion in the electrode materials. The relationships between *Z*′ and *ω*^−1/2^ in the frequency region of 0.1–0.01 Hz are shown in Fig. S7b, *Z*′ is the Warburg impedance and *ω* is the angular frequency in the low-frequency region. Generally, the smallest Warburg impedance coefficient *σ* value is, the largest diffusion coefficient D will be. By comparing the line slopes between *Z*′ and *ω*^−1/2^ of the MgVO cathode materials in the five different electrolytes (Fig. S7b), the smallest slope of the line indicated the improved the ion permeability and electron conductivity. By fitting the AC impedance spectrum, the detailed calculation results of the parameters are listed in Table S2. The diffusion coefficient *D* of the electrode in 1.0Zn1.0Mg electrolyte is calculated to be 1.21 × 10^−14^ cm^2^ s^−1^, larger than that of the other four systems, indicating that the 1.0Zn1.0Mg system has distinctly faster charge transfer kinetics. The above results indicate that the appropriate pre-addition of magnesium ions can provide a continuous and fast pathway for electron transport.

To further understand the good rate performance of MgVO cathode performed in the hybrid 1.0 M ZnSO_4_–1.0 M MgSO_4_ electrolyte, the electrochemical kinetic characteristic of the ion intercalation/deintercalation was investigated and the capacitive contribution was further quantitatively determined using previously reported CV characterizations (see details in the Supplementary Note).

To quantitatively distinguish the diffusion-controlled contribution and capacitive contribution to current, CV curves at various sweep rates from 0.1 to 0.5 mV s^−1^ are recorded in Fig. 4g, with a voltage window from 0.2 to 1.4 V. Figure 4g displays two reduction peaks (A, C) and two oxidation peaks (B, D) in each CV curve. When the scan rates increased, the current densities increased correspondingly and the redox peaks are even more obvious, but the anodic and cathodic peaks did not show obvious shifts toward positive/negative potential, indicating that the process of ion insertion/deinsertion might be highly reversible.

By fitting the slope of log(*i*) versus log(*v*) plots (Fig. 4h), the *b* values of the redox peak A, B, C, and D are calculated to be 0.93, 0.84, 0.81, and 0.87, respectively, suggesting that the pseudocapacitive-like kinetics hold the dominant position. Furthermore, the capacitive contribution is also calculated to be 68.9% of the total current (namely, the capacity) at the scan rate of 0.1 mV s^−1^, indicating that the electrochemical reaction is controlled by ionic diffusion as well as pseudocapacitance and the capacitive contribution is dominated in the total capacity. With the increase in sweep rate, the ratio of capacitive to diffusive current gradually raises to 73.0%, 75.9%, 78.0%, and 81.0% at the scan rates of 0.2, 0.3, 0.4, and 0.5 mV s^−1^, respectively (Fig. 4h), revealing that the Zn/MgVO batteries have favorable charge transfer kinetics which is responsible for the high rate performance.

### Electrochemical Reaction Mechanism of the MgVO Electrodes

**Fig. 5:**
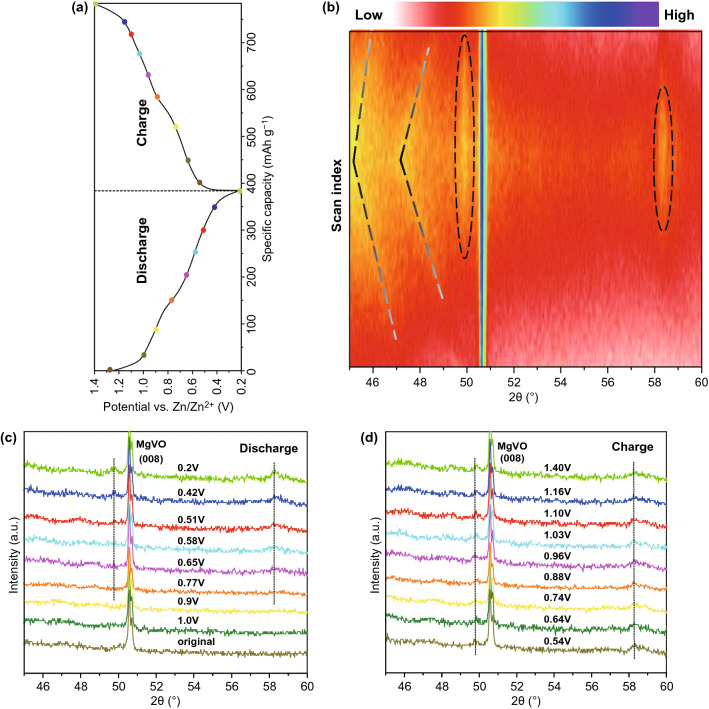
The first discharge–charge curve (**a**), the corresponding operando XRD patterns in a contour plot (**b**) and the selective XRD patterns at different stages of discharge/charge (**c**, **d**) obtained at the current density of 100 mA g^−1^ in 1.0 M ZnSO_4_–1.0 M MgSO_4_ electrolyte within a potential window from 0.2 to 1.4 V

In order to establish the detailed structure transformation and electrochemical behavior during the electrochemical reaction, in situ XRD was conducted during the first discharge–charge process to reveal the evolution of the peak intensities [33, 34]. Figure 5a illustrates the first discharge–charge profile for the MgVO electrode in hybrid 1.0Zn1.0Mg electrolyte with the selected operando XRD scan voltages, while a 2D mapping contour plot with XRD patterns in the selected angle region is displayed in Fig. 5b–d. During the in situ XRD measurement process, the diffraction peaks come from the current collector; cell components of stainless-steel Swagelok shells and materials for X-ray windows are mostly inevitable. Therefore, a proper diffraction window should be precisely chosen before the operando testing. Herein, the strongest (001) peak of the MgVO located at 2*θ* = 6.34° is overlapped by the peak from the cell components, and the second strong peak of the MgVO located at 2*θ* = 27° is overlapped by the peak from the current collector of carbon paper. As a result, the selected XRD 2θ scan range is chosen from 45° to 60° for the best display of the in situ XRD measurement.

During the cycling process, two pronounced shifts of the contour plots to lower scanning angles are observed, due to the continuous ion intercalation and lattice spacing expansion (Fig. 5b). The position of the two new peaks becomes more negative shift as the intercalation proceeding and continues to shift toward the lowest angle at the end of discharge, followed by the corresponding diffraction positions recover back to higher scanning angles with the intensity becoming weaker gradually as the deintercalation progress (marked with dashed crease lines in Fig. 5b). The negative shifts to lower angles of diffraction peaks along with the intercalation (discharge) process have also been observed for the sodium vanadate [35]. According to the Bragg equation, the negative shifts could be explained by the distance expanding of these crystal faces, which results from the insertion of Zn^2+^ ions. This expansion of interlayer space is derived from the reduction of V^5+^ ions and thereby increases the M–M bond distance between the layers [17, 32, 35]. And then in the charge process, since the vanadium is oxidized to the initial state, the diffraction peaks gradually recover back to the original angles when the voltage was raised, indicating the excellent reversible process of Zn^2+^ ion intercalation/deintercalation. However, the negative shift mentioned above cannot be observed notably in the XRD patterns, which might attribute to the weak and broad characteristic of the reflections. And it is also difficult to observe the initial reflections in the commencing few scans, which makes it harder to identify the specific phase.

As shown in the extracted XRD patterns (Fig. 5c, d), only a prominent sharp peak located at 2*θ* = 50.6° is observed and seems to maintain the peak intensities all along without shifting for the whole testing procedure, which is corresponded to the (008) peak from the MgVO nanobelts. However, the (115) peak from the MgVO is not shown in Fig. 5, which might be ascribed to two reasons: on the one hand, the (115) peak itself has relatively low intensity and is originally unobvious; on the other hand, the specialized setup of the Swagelok-type cell for the in situ XRD measurements with polymide as X-ray window material and carbon paper as current collector has greatly blocked the signals. With an increase in the discharge depth, two new weak and broad peaks around 2*θ* = 49.8° and 2*θ* = 58.3° gradually form (marked and circled with dashed lines), corresponding to the formation of Zn_*x*_V_2_O_5_ [11]. The intensity of the two new peaks becomes stronger with zinc-ion content as the intercalation proceeds and increases to the strongest intensity at the end of discharge. During the subsequent deintercalation process until the end of complete charge reaction, the new peaks have a reasonable decrease in the intensities.

To gain insight into the structural and morphological changes, the MgVO electrodes cycled in the 1.0 M ZnSO_4_–1.0 M MgSO_4_ electrolyte at different discharge/charge states were collected and studied from the disassembled cells. The cycled MgVO electrodes collected at the fully second discharged, the fully second charged, and the 100th cycle charged states are investigated by the ex situ XRD, XPS, EDS, SEM, and TEM characterizations.

After the first cycle, the Mg element could be barely seen in the MgVO electrode material when secondly discharging to 0.2 V, a considerable amount of Zn is found instead, and the Mg/Zn/V ratio is determined to be ~ 0.07:1.54:2 by SEM–EDS elemental mapping (Fig. S8a-d) and TEM-EDS. The result indicates that the Zn^2+^ ion intercalation plays a dominant role in the specific capacity contribution. When secondly charging to 1.4 V, the Mg/Zn/V ratio is determined to be ~ 0.04:0.36:2 by SEM–EDS elemental mapping (Fig. S9a-d) and TEM-EDS. This result suggests that most of the Mg^2+^ ions have been deintercalated and displaced by Zn^2+^ ions to form Zn*z*Mg*x*V_2_O_5_ serving as the cathode material after the first cycle. Such a phenomenon has also been observed in Ag_0.4_V_2_O_5_ [14] and Na_3_V_2_(PO_4_)_3_ [19] for Zn-ion batteries with the displacement/intercalation reaction mechanism [14].

From the TEM images in Figs. S8e and S9e, it can be seen that the distinct outlines of the MgVO nanobelts have been better maintained, with the surface of the nanobelts becoming rough. The HRTEM images display the marked lattice fringe with spacing of 0.206 nm (Fig. S8f, discharged) and 0.210 nm (Fig. S9f, charged), which are larger than the spacing of (006) plane for MgVO. These results are also confirmed by the XRD (Fig. S10) and XPS (Fig. S11) studies. At the secondly full discharge state, a newly emerged phase has been observed upon discharging to 0.2 V (Fig. S10a). Unfortunately, the new specific compound is hard to identify because the co-intercalation process is complicated. After secondly charging to 1.4 V (Fig. S10b), another new phase can be indexed to zinc pyrovanadate (Zn_3_(OH)_2_V_2_O_7_·2H_2_O, JCPDS No. 50-0570), which further confirms the displacement/intercalation reaction mechanism [14].

The ex situ XPS analyses of cycled MgVO electrodes at different discharge/charge states are further performed. As depicted in Fig. S11a, there is no signal in the Zn 2*p* region in the pristine material that can be detected. After the first cycle, the Zn^2+^ ions have been successfully inserted to form the new Zn-containing compound, as a result, there are sharp and strong Zn 2*p* peaks emerging both in the fully second discharged (Fig. S11c) and the fully second charged (Fig. S11e) states. Specifically, in the fully discharged state (Zn^2+^ insertion), two more Zn 2*p* features are newly evolved except for the indigenous Zn 2*p* peaks (2*p*_3/2_: 1021.8 eV; 2*p*_1/2_: 1044.9 eV), implying the repeated intercalation/deintercalation processes of Zn^2+^ ions (Fig. S11c) [11].

Furthermore, the reversible electrochemical reduction of the V–O–V framework as a consequence of Zn^2+^ (de)intercalation is confirmed by the evolution of V 2*p* XPS peaks. As shown in Fig. S11b, d, f, the peaks around binding energies of 524 and 517 eV can be assigned to the V^5+^ 2*p*_1/2_ and V^5+^ 2*p*_3/2_ peaks, respectively. There will gradually involve some V^4+^ species (V^4+^ 2*p*_3/2_: 516.3 eV; V^4+^ 2*p*_1/2_: 523.8 eV) upon the repeated discharge/charge processes. Additionally, the discharged cathode analysis reveals an intensification in V^4+^ signals and a decrease in V^5+^ peaks associated with the Zn^2+^ intercalation and consequent reduction of the V–O–V framework (Fig. S11d). After that, the V^5+^ species again becomes the dominant features upon the charging process (Fig. S11f).

The MgVO cathode is further investigated when cycling at the current density of 1 A g^−1^ for 100 cycles in the 1.0 M ZnSO_4_–1.0 M MgSO_4_ electrolyte. The XRD pattern (Fig. S12a) reveals that the new phase of the Zn_3_(OH)_2_V_2_O_7_·2H_2_O has gradually increased, compared to that of the secondly charged cycle (Fig. S10b). As shown in Fig. S12b, an intensity decrease for Mg 1 *s* can be observed in the cycled state, comparing to the initial state of the pristine material (Fig. S2a). The Zn 2*p* region (Fig. S12c) and the V 2*p* region (Fig. S12d) are similar to that of the second fully charged state (Fig. S11e, f), however, partial intercalated Zn^2+^ ions cannot be completely extracted. Moreover, the SEM (Fig. S12e) and TEM (Fig. S12f) images of the electrodes after 100 cycles reveal that the nanobelt morphology has been well preserved.

**Fig. 6:**
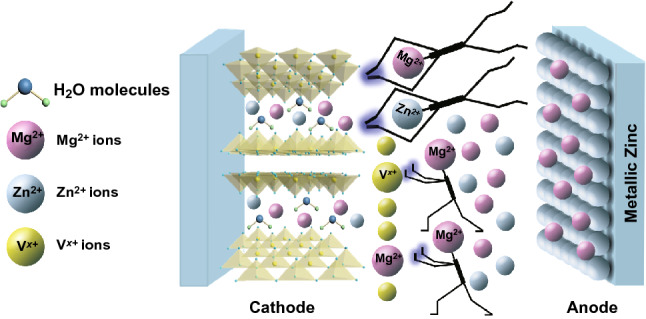
Scheme of the Mg^2+^ functional mechanism for hybrid electrolyte

The mechanism of the Mg^2+^ ions as inorganic additives in the aqueous ZIBs then can be concluded, which can be explained from two aspects, as shown in Fig. 6. On the one hand, the addition of Mg^2+^ into the electrolyte will change the dissolution equilibrium of Mg^2+^ from the MgVO cathode and thus impede the continuous dissolution of the active materials, thus suppress the rapid degradation in capacity. The inserted Mg^2+^ ions between the V_x_O_y_ layers can act as “pillars” and play an important role to maintain the structural stability of the MgVO during the discharge–charge process. This explanation was supported by the observation of the color changes of the electrolytes (Fig. S13), in which the MgVO electrodes dipping into the 0.5 M ZnSO_4_–1.5 M MgSO_4_ and 2.0 M MgSO_4_ electrolytes for 24 h were kept transparent and colorless, indicating that the dissolution of MgVO was successfully suppressed. By contrast, the MgVO electrode dipping into the 2 M ZnSO_4_ electrolyte showed an obvious yellow, probably derived from the color of VO^2+^ ions [25]. On the other hand, both Zn^2+^ and Mg^2+^ ions could both insert into the layered structure of MgVO during discharging in the Zn^2+^/Mg^2+^ containing solution, which contributes to the larger capacities. Mg^2+^ ions are demonstrated to serve as the co-insertion cations along with the Zn^2+^ ions upon discharge, then the deintercalated Mg^2+^ ions dissolve back in the electrolyte and deposit on the Zn anode upon charging process [13]. The Zn anodes cycled in the 1.0 M ZnSO_4_–1.0 M MgSO_4_ electrolyte at the fully discharged and the fully charged states were collected from the disassembled cells and investigated by the SEM–EDS elemental mapping characterization (Fig. S14). The Mg^2+^ ions are found to deposit on the Zn plate anode upon the charging process, as shown in Fig. S14f, the SEM–EDS elemental mapping data reveals the distribution of magnesium element on the Zn plate surface and detects a 0.29 wt% of Mg deposition. These Mg^2+^ ions will then dissolve back in the electrolyte and serve as the interaction cations along with the Zn^2+^ ions upon the discharging process, with scarcely any Mg distribution on the Zn anode (0.01 wt%, Fig. S14c).

## Conclusions

A facile one-pot synthesis of Mg_x_V_2_O_5_·nH_2_O nanobelts has been developed, and its application as cathode materials in rechargeable aqueous ZIBs has also been demonstrated. The MgVO cathodes were investigated in five electrolytes with different concentration ratios of ZnSO_4_ and MgSO_4_, i.e., 2.0 M ZnSO_4_, 1.5 M ZnSO_4_–0.5 M MgSO_4_, 1.0 M ZnSO_4_–1.0 M MgSO_4_, 0.5 M ZnSO_4_–1.5 M MgSO_4_, and 2.0 M MgSO_4_ electrolytes. Comparing the electrochemical performance performed in five electrolytes through CV and galvanostatic discharge/charge measurements, it is found that the electrochemical behaviors gradually change when increasing the MgSO_4_ concentration in the aqueous electrolytes. Among the MgVO cathodes measured in five electrolytes, the one in the electrolyte of 1.0 M ZnSO_4_–1.0 M MgSO_4_ obtains the best cycling and rate performance results, which delivers a high specific capacity of 374 mAh g^−1^ at a current density of 100 mA g^−1^, maintains a retention of 90.3% at a current density of 1 A g^−1^ for 200 cycles, and exhibits a reversible capacity of 175 mAh g^−1^ at 5 A g^−1^. Our studies put a light upon the way to explore the cost-effective electrolytes for the stationary grid-scale applications.

## Electronic Supplementary Material

Below is the link to the electronic supplementary material.

Supplementary material 1 (PDF 1713 kb)
