# Supplementary material for: Rechargeable Aqueous Zinc-Ion Batteries in MgSO4/ZnSO4 Hybrid Electrolytes
Source: Nanomicro Lett. 2020 Feb 21;12:60. doi: 10.1007/s40820-020-0385-7 (PMC7770650; doi:10.1007/s40820-020-0385-7)
Supplement: Supplementary file 1 — Supplementary material 1 (PDF 1713 kb) [file 40820_2020_385_MOESM1_ESM.pdf]

Supporting Information for

Rechargeable Aqueous Zinc-Ion Batteries in $MgSO_4$/$ZnSO_4$ Hybrid Electrolytes

Yingmeng Zhang[1, 2, 3], Henan Li[4, *], Shaozhuan Huang[5], Shuang Fan[5], Lingna Sun[1], Bingbing Tian[2], Fuming Chen[6], Ye Wang[7], Yumeng Shi[2, 3, *], Huiying Yang[5, *]

[1]College of Chemistry and Environmental Engineering, Shenzhen University, Shenzhen, Guangdong 518060, People's Republic of China

[2]International Collaborative Laboratory of 2D Materials for Optoelectronics Science and Technology of Ministry of Education, Institute of Microscale Optoelectronics, Shenzhen University, Shenzhen 518060, People's Republic of China

[3]Engineering Technology Research Center for 2D Material Information Function Devices and Systems of Guangdong Province

[4]College of Electronic Science and Technology, Shenzhen University, Shenzhen 518060, People's Republic of China

[5]Pillar of Engineering Product Development, Singapore University of Technology and Design, 8 Somapah Road, 487372, Singapore

[6]School of Physics and Telecommunication Engineering, South China Normal University, Guangzhou 510006, People's Republic of China

[7]Key Laboratory of Material Physics of Ministry of Education, School of Physics and Engineering, Zhengzhou University, Zhengzhou 450052, People's Republic of China

*Corresponding authors. E-mail: yanghuiying@sutd.edu.sg (Huiying Yang), yumeng.shi@szu.edu.cn (Yumeng Shi), henan.li@szu.edu.cn (Henan Li)

S1 Supplementary Note

S1.1 Estimation of the Capacitive Contribution and Diffusion-controlled Contribution

Figure 4g in the main text displays two reduction peaks and two oxidation peaks in each CV curve.

Generally, the measured peak currents (i) and scan rates (v) have a relationship follows the power law as below:

$$i = av^b$$

which can be rewritten as,

$$\log(i) = \log(a) + b \cdot \log(v)$$

Therefore, the b value can be acquired by fitting slope of the log(i) versus log(v) profile. The b value is often in a range of 0.5–1.0, where a b value tends to 0.5 indicates the electrochemical process is controlled by ionic diffusion, while the b value reaches to 1.0 corresponds to the capacitive dominated behavior.

Assuming the current response (i) depends on a combination of the pseudocapacitance and diffusion-controlled process, the capacitive contribution can be further quantitatively calculated according to the following equation:

$$i = k1v + k2v^{1/2}$$

which can be reformulated as,

$$i/v^{1/2} = k1v^{1/2} + k2$$

where $k1v$ and $k2v^{1/2}$ represent the capacitive and ionic diffusion contribution, respectively.

The capacitive contribution can be estimated by determining the $k1$ value, which can be obtained by fitting the $i/v^{1/2}$ vs. $v^{1/2}$ plots.

S1.2 Estimation of the Diffusion Coefficient Values Calculated for the MgVO Cathodes in the Five Different Electrolytes

The diagonal lines in the low-frequency region presented in the EIS results are assigned to the ion diffusion in the electrode materials. The diffusion coefficient values can be quantitatively calculated by Eqs. S1 and S2:

$$Z' = R_D + R_L + \sigma\omega^{-1/2} \qquad \text{(S1)}$$

$$D = \frac{R^2T^2}{2A^2n^4F^4C^2\sigma^2} \qquad \text{(S2)}$$

In Eq. S1, Z' is defined to be the Warburg impedance, ω is the angular frequency in the low frequency region, R_D is the diffusion resistance, and R_L is the liquid resistance. And the Warburg impedance coefficient (denoted by σ) is the key factor what we want to obtain, which is calculated by the slope of the oblique line corresponding to the relationships between Z' and $\omega^{-1/2}$ in the low frequency region. In the Eq. S2, D denotes diffusion coefficient, R is the gas constant, T is denoted as the thermodynamic temperature, A is the effective contract area of the electrode, F is the Faraday constant, and C is the concentration of the active ions.

S2 Supplementary Figures and Tables

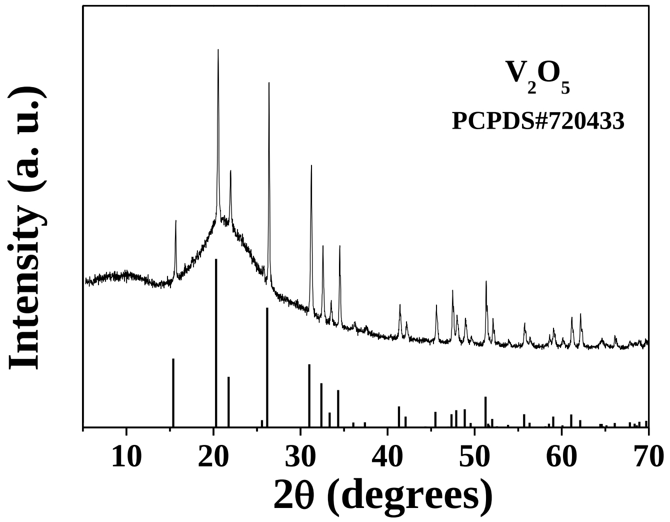

Fig. S1 The XRD pattern of the commercial V_2O_5 reagent

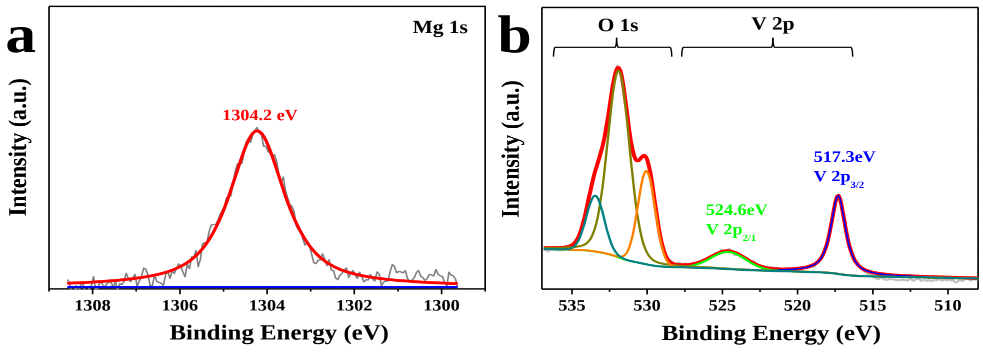

Fig. S2 The XPS spectra of the MgVO material

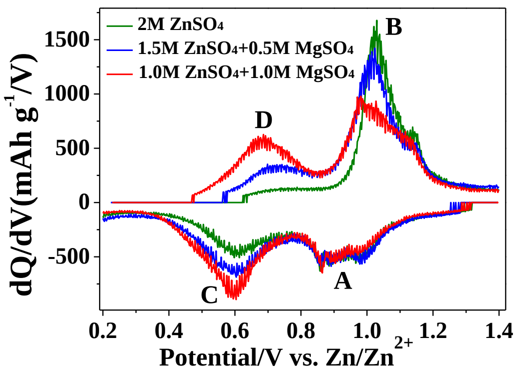

Fig. S3 The corresponding derivative dQ/dV response derived from the discharge–charge profiles of MgVO cathode at current density of 100 mA g^{-1} in different electrolytes

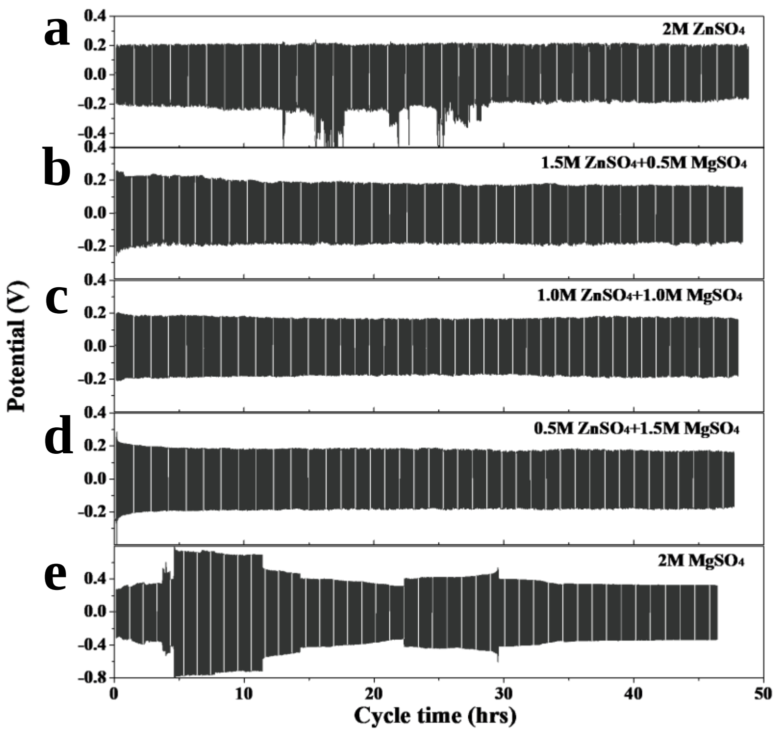

Fig. S4 Voltage profiles as a function of time for zinc plating/stripping experiments in symmetric Zn/electrolyte/Zn cells cycled at a current density of 10 mA/cm^2 for 5000 cycles, the electrolytes are (**a**) 2.0 M $ZnSO_4$, (**b**) 1.5 M $ZnSO_4$–0.5 M $MgSO_4$, (**c**) 1.0 M $ZnSO_4$–1.0 M $MgSO_4$, (**d**) 0.5 M $ZnSO_4$–1.5 M $MgSO_4$, and (**e**) 2.0 M $MgSO_4$ electrolytes

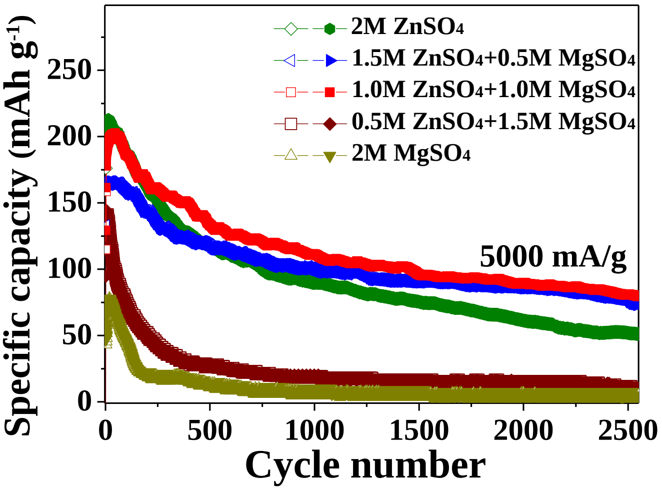

Fig. S5 Prolonged cycling performance of the MgVO cathodes in five different electrolytes of 2Zn0Mg, hybrid 1.5Zn0.5Mg, hybrid 1.0Zn1.0Mg, hybrid 0.5Zn1.5Mg and 0Zn2Mg, respectively, with the current density of 5000 mA g^{-1}

After 2500 cycles at a current density of 5000 mA g^{-1}, it can be observed that a relatively good cycling stability of the MgVO cathode is measured in the 2Zn0Mg, 1.5Zn0.5Mg and 1.0Zn1.0Mg electrolytes, while the testing in the 0.5Zn1.5Mg and 0Zn2Mg electrolytes shows an obvious capacity fading. Among them, the electrode in the 1.0Zn1.0Mg electrolyte exhibits the slowest capacity fading.

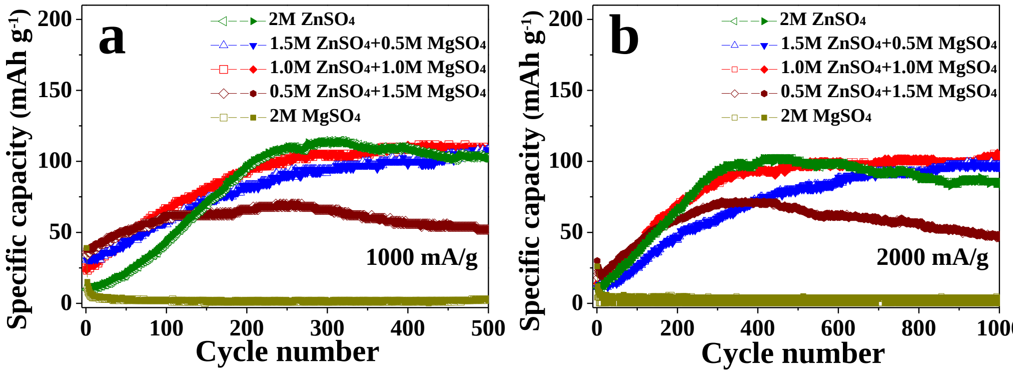

Fig. S6 Cycling performance of the commercial V_2O_5 powder cathodes in five different electrolytes of 2Zn0Mg, hybrid 1.5Zn0.5Mg, hybrid 1.0Zn1.0Mg, hybrid 0.5Zn1.5Mg and 0Zn2Mg, respectively, with the current density of 1000 mA g^{-1} (**a**) and 2000 mA g^{-1} (**b**)

As shown in **Fig. S6,** it can be observed that the specific capacities increase over several hundred cycles until to a maximum value except for the cathode in the 2.0 M $MgSO_4$ electrolyte, indicating that a gradual activation is highly required to improve the kinetics and the discharge capacity can then be fully displayed. The cathodes in the hybrid 1.5Zn0.5Mg and hybrid 1.0Zn1.0Mg electrolytes show a stable cycling performance after the electrode activation. By contrast, the cathode in the 2.0 M $ZnSO_4$ electrolyte presents the gradually decreased capacities.

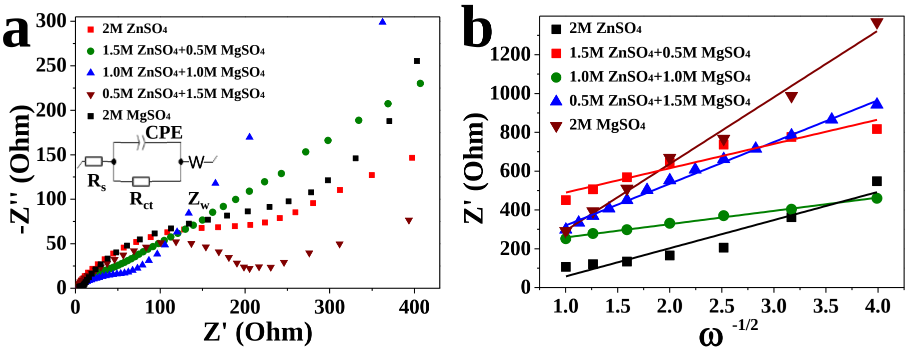

Fig. S7 (**a**) Nyquist plots of the MgVO electrodes in five different electrolytes, which were carried out in the frequency range of 0.01−100kHz. The inset shows the corresponding equivalent EIS circuitry model. (**b**) The line relationship of the cathodes in five electrolytes between Z' and $\omega^{-1/2}$ in the frequency region of 0.1-0.01 Hz

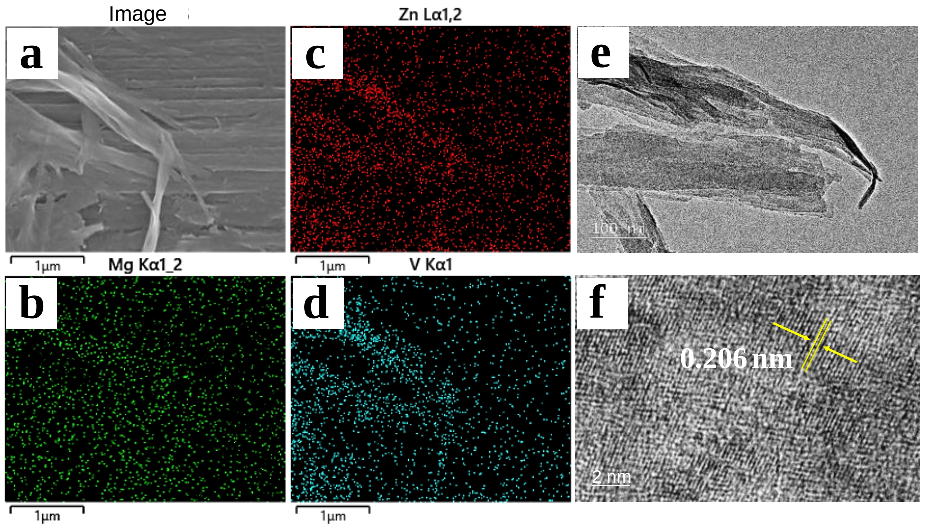

Fig. S8 Characterization of the MgVO cathode at the fully second discharged state when cycling at current density of 100 mA g^{-1} in the 1.0 M $ZnSO_4$–1.0 M $MgSO_4$ electrolyte: (**a-d**) SEM-EDS elemental mapping images, (**e**) TEM image, (**f**) the corresponding HRTEM image

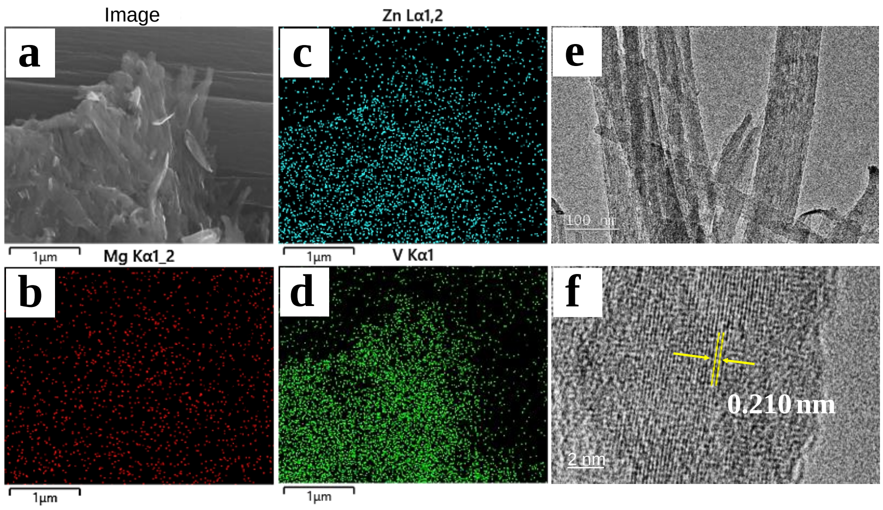

Fig. S9 Characterization of the MgVO cathode at the fully second charged state when cycling at current density of 100 mA g^{-1} in the 1.0 M $ZnSO_4$–1.0 M $MgSO_4$ electrolyte: (**a-d**) SEM-EDS elemental mapping images, (**e**) TEM image, (**f**) the corresponding HRTEM image

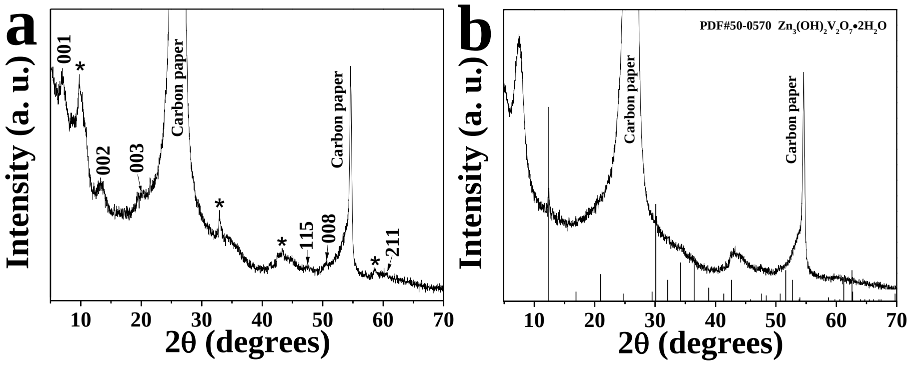

Fig. S10 XRD patterns of the MgVO cathode at the fully second discharged (**a**) and charged (**b**) state when cycling at current density of 100 mA g^{-1} in the 1.0 M $ZnSO_4$–1.0 M $MgSO_4$ electrolyte

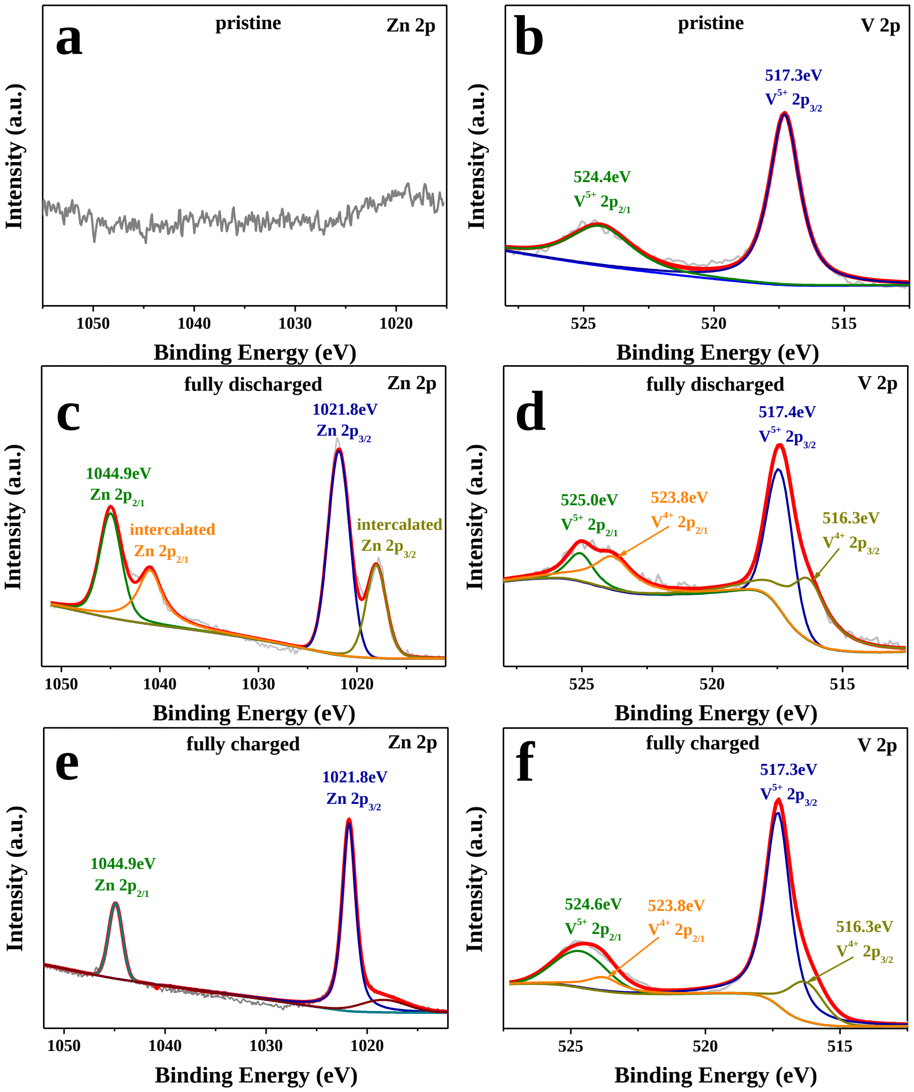

Fig. S11 XPS spectra of the MgVO cathode cycled at current density of 100 mA g^{-1} in the 1.0 M $ZnSO_4$–1.0 M $MgSO_4$ electrolyte: Zn 2*p* region of the XPS spectra (**a, c, e**), and V 2*p* region of the XPS spectra (**b, d, f**). Pristine material (**a, b**), the fully discharged electrode (**c, d**) and the fully charged electrode (**e, f**)

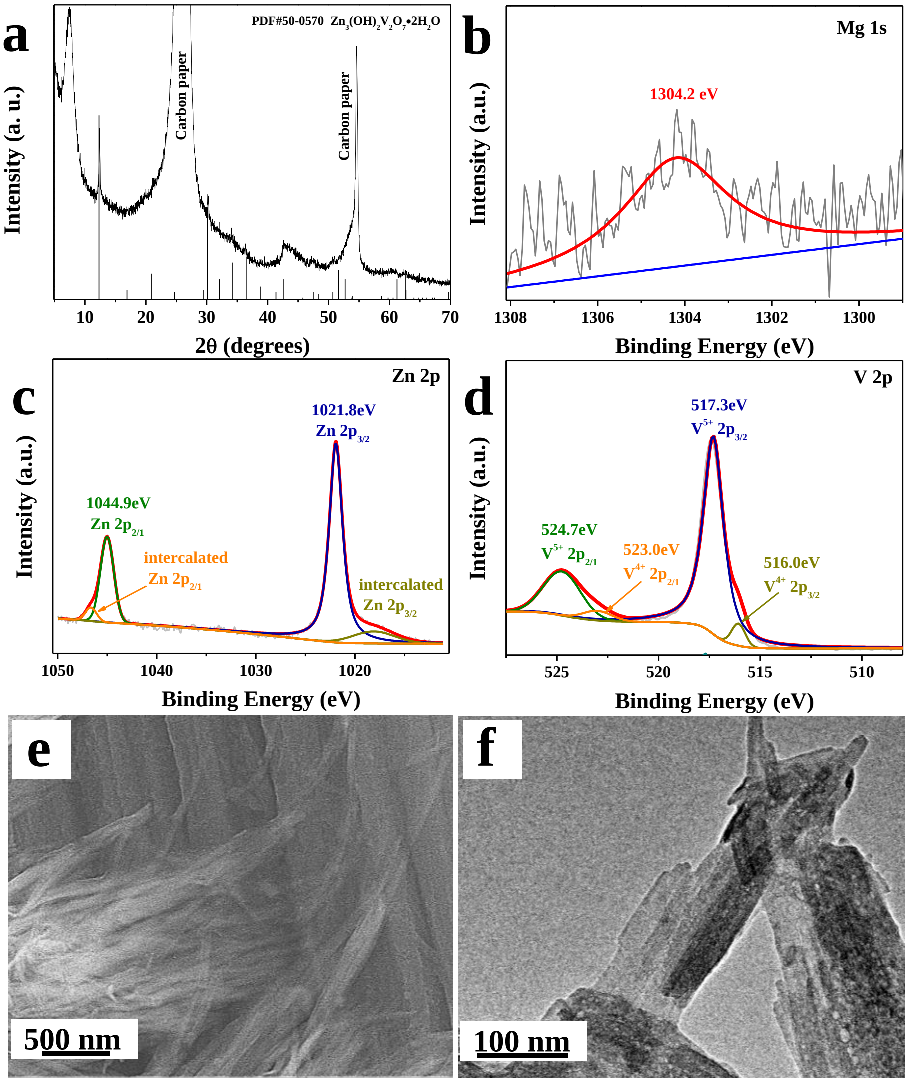

Fig. S12 (**a**) XRD pattern, (**b-d**) XPS spectra, (**e**) SEM image and (**f**) TEM image of the MgVO cathode at the 100th cycle charged state when cycling at the current density of 1 A g^{-1} for 100 cycles in the 1.0 M $ZnSO_4$–1.0 M $MgSO_4$ electrolyte

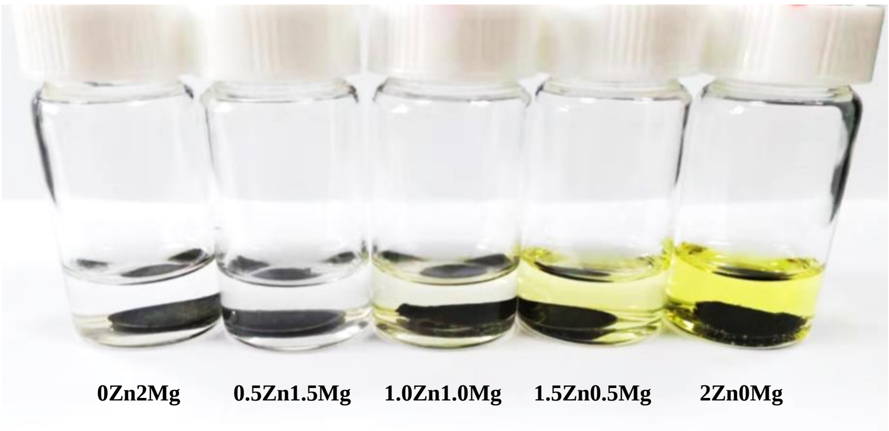

Fig. S13 Optical image of the MgVO electrodes in different electrolytes for 24 h. With the increase of $MgSO_4$ concentration, the dissolution of active material is effectively controlled

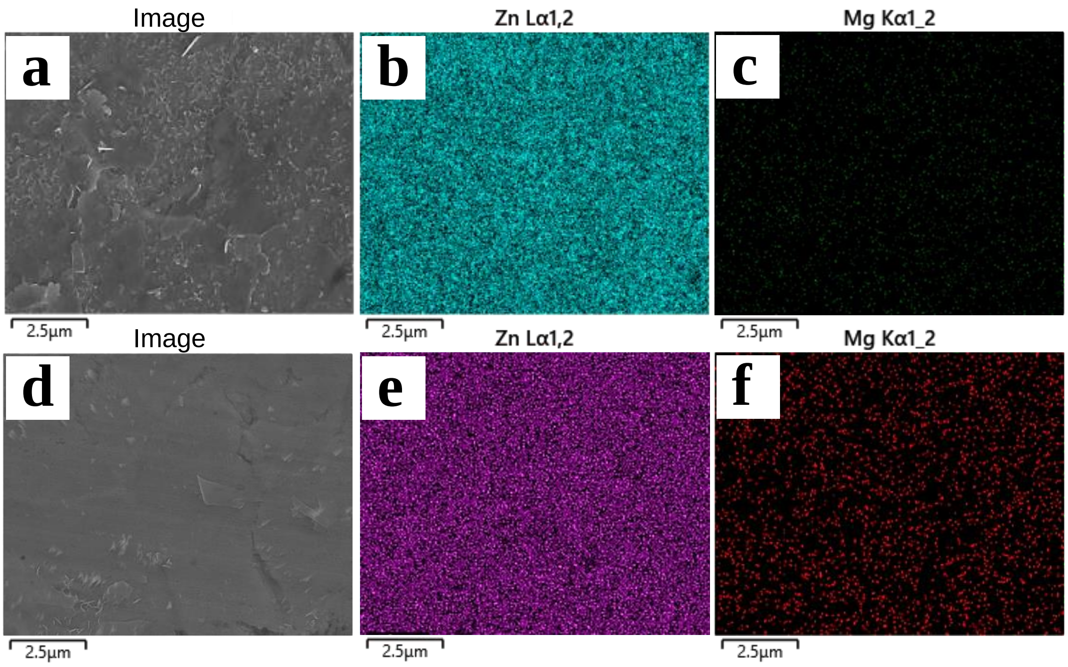

Fig. S14 SEM-EDS elemental mapping images of the Zn anodes at the fully discharged (**a-c**) and charged (**d-f**) states when cycling at current density of 100 mA g^{-1} in the 1.0 M $ZnSO_4$–1.0 M $MgSO_4$ electrolyte

Table S1 Comparison of electrochemical properties of MgVO with previously reported metal vanadate cathode materials for aqueous ZIBs

**Metal vanadates**	**Electrolytes**	**Rate performance**	**Cycling performance**	**References**
$Mg_xV_2O_5{\cdot}nH_2O$	1.0 M $ZnSO_4$–1.0 M $MgSO_4$	374, 330, 280, 241, 216, and 175 mA h g^{-1} at 0.1, 0.2, 0.5, 1, 2, and 5 A g^{-1}, respectively	90% after 200 cycles at 1 A g^{-1}<br>72% after 600 cycles at 2 A g^{-1}	This work
$Mg_{0.34}V_2O_5{\cdot}nH_2O$	3M $Zn(CF_3SO_3)_2$	353, 330, 291, 264, 221, 167, and 81 mA h g^{-1} at 0.05, 0.1, 0.5, 1, 2, 3, and 5 A g^{-1}, respectively	100% after 200 cycles at 0.1 A g^{-1}<br>95% after 1000 cycles at 1 A g^{-1}<br>97% after 2000 cycles at 5 A g^{-1}	ACS Energy Letters 2018, 3, 2602
$Na_2V_6O_{16}{\cdot}1.63H_2O$	3M $Zn(CF_3SO_3)_2$	352, 335, 316, 261, 219, and 162 mA h g^{-1} at 0.05, 0.1, 0.2, 0.5, 1, and 2 A g^{-1}, respectively	78% after 100 cycles at 0.1 A g^{-1}<br>75% after 500 cycles at 1 A g^{-1}<br>90% after 6000 cycles at 5 A g^{-1}	Nano Lett. 2018, 18, 1758
$Ca_{0.24}V_2O_5{\cdot}nH_2O$	1 M $ZnSO_4$	~200, ~150, and 72 mA h g^{-1} at 10 C, 30C and 80C, respectively (1C=250 mA g^{-1})	96% after 3000 cycles at 80 C<br>78% after 5000 cycles at 80 C	Angew. Chem. Int. Ed. 2018, 57, 3943

$Zn_3V_2O_7(OH)_2{\cdot}2H_2O$	1 M $ZnSO_4$	200, 166, 145, 122, 105, 84, 75, and 54 mA h g^{-1} at 0.05, 0.1, 0.3, 0.5, 0.8, 1, 2, and 3 A g^{-1}, respectively	68% after 300 cycles at 0.2 A g^{-1}	Adv. Mater. 2018, 30, 1705580
$Ag_{0.4}V_2O_5$	3 M $ZnSO_4$	No accurate values recorded in the text	216 mA h g^{-1} after 1000 cycles at 5 A g^{-1} 155 mA h g^{-1} after 2000 cycles at 10 A g^{-1}	Energy Storage Mater. 2018
$Zn_{0.25}V_2O_5{\cdot}nH_2O$	1 M $ZnSO_4$	~260, 223 and 183 mA h g^{-1} at 8C, 15 C, and 20 C, respectively (1C=300 mA g^{-1})	81% after 1000 cycles at 8 C	Nature Energy 2016, 1, 16119
LiV_3O_8	1 M $ZnSO_4$ (pH 4.0)	188, 148, 79, 47, and 29 mA h g^{-1} at 0.133, 0.266, 0.533, 1.066, and 1.666 A g^{-1}, respectively	90 % after 65 cycles at 0.133 A g^{-1}	Chem. Mater. 2017, 29, 1684

Table S2 Calculated R_{ct}, σ and D values from the EIS results for the cathodes in five electrolytes

**Electrolytes**	**R_{ct}（Ω）**	**σ（Ω cm^2 $s^{-0.5}$）**	**D（cm^2 s^{-1}）**
2.0 M $ZnSO_4$	275	145.24	2.68×10^{-15}
1.5 M $ZnSO_4$–0.5 M $MgSO_4$	78	125.4	3.60×10^{-15}
1.0 M $ZnSO_4$–1.0 M $MgSO_4$	106	68.425	1.21×10^{-14}
0.5 M $ZnSO_4$–1.5 M $MgSO_4$	214	215.06	1.22×10^{-15}
2.0 M $MgSO_4$	334	343.55	4.79×10^{-16}

Note: R_{ct} represents the resistance of the charge during the transfer process in the electrode material from the equivalent circuit.
